# The effect of New Neonatal Porcine Diarrhoea Syndrome (NNPDS) on average daily gain and mortality in 4 Danish pig herds

**DOI:** 10.1186/1746-6148-10-90

**Published:** 2014-04-22

**Authors:** Hanne Kongsted, Helle Stege, Nils Toft, Jens P Nielsen

**Affiliations:** 1 Danish Pig Research Centre, Danish Agriculture and Food Council, Vinkelvej 13, Kjellerup 8620, Denmark; 2 HERD – Centre for Herd-oriented Education, Research and Development, Department of Large Animal Sciences, University of Copenhagen, Groennegaardsvej 2, Frederiksberg C 1870, Denmark; 3 National Veterinary Institute, Technical University of Denmark, Bülowsvej 27, Frederiksberg C 1870, Denmark

## Abstract

**Background:**

The study evaluated the effect of New Neonatal Porcine Diarrhoea Syndrome (NNPDS) on average daily gain (ADG) and mortality and described the clinical manifestations in four herds suffering from the syndrome. NNPDS is a diarrhoeic syndrome affecting piglets within the first week of life, which is not caused by enterotoxigenic *Escherichia coli* (ETEC), *Clostridium perfringens* (*C. perfringens*) type A/C, *Clostridium difficile* (*C. difficile*), rotavirus A, coronavirus, *Cystoisospora suis*, *Strongyloides ransomi*, *Giardia spp* or *Cryptosporidium spp*.

**Results:**

Piglets were estimated to have a negative ADG of 9 and 14 g when diarrhoeic for 1 day and >1 day respectively. However, if only diarrhoeic on the day of birth, no negative effect on ADG was seen. Piglets originating from severely affected litters were estimated to have a reduced ADG of 38 g. The study did not show an overall effect of diarrhoea on mortality, but herd of origin, sow parity, birth weight, and gender were significantly associated with mortality. In one of the herds, approximately 25% of the diarrhoeic piglets vs. 6% of the non-diarrhoeic piglets died, and 74% of necropsied piglets were diagnosed with enteritis. These findings indicate that the high mortality seen in this herd was due to diarrhoea.

**Conclusions:**

NNPDS negatively affected ADG in piglets, and even piglets that were diarrhoeic for one day only experienced a reduction in ADG. However, the study showed that diarrhoea restricted to the day of birth did not affect ADG and suggested this phenomenon to be unrelated to the syndrome. Since the diarrhoeal status of the litter had important effects on ADG, future research on NNPDS probably ought to focus on piglets from severely affected litters.

The study showed important dissimilarities in the course of diarrhoea between the herds, and one herd was considerably more affected than the others. Within this herd, NNPDS seemed to be associated with a higher mortality, whereas in general the study did not show lethal effects of NNPDS.

## Background

Several researchers have suggested the emergence of a new neonatal diarrhoeal syndrome [1-5]. The present study is the second in a series from our research group studying this new syndrome. All studies were carried out in the same four herds having a long history of unexplained neonatal diarrhoea. Running parallel to the current study, a microbiological study was carried out. The results of the microbiological study suggested that neither ETEC, *C. perfringens* type A/C, *C. difficile*, rotavirus A, coronavirus, *Cystoisospora suis*, *Strongyloides ransomi*, *Giardia spp* nor *Cryptosporidium spp* were associated with the syndrome [6].

Previous studies on the effect of diarrhoea in suckling pigs estimated reductions in ADG of 8–14 g per day [7,8]. Various studies have estimated significant associations between suckling pig diarrhoea and mortality [9,10] or diagnosed enteritis as an important cause of death in the suckling period [11-13].

The primary objective of the current study was to evaluate the effect of NNPDS on average daily gain (ADG) and mortality in relation to timing and duration of clinical symptoms in individual piglets. Effects were estimated with respect to individual symptoms as well as symptoms at litter level.

The secondary objective was to describe the course of disease in herds suffering from NNPDS.

## Results

In total, 989 piglets were ear tagged at birth. During the study period, 110 piglets were euthanized for diagnostic purposes (for results: See [6]) and five were excluded due to hermaphroditism or missing data. This left 874 piglets (207, 228, 202 and 237 from herds 1, 2, 3 and 4, respectively) for the study on mortality.

During the study period 80 piglets died, hence could not be included in the study on ADG. Furthermore, for this part of the study a critical limit on five piglets per litter was set resulting in exclusion of 14 piglets (the remaining part of four litters). In total, 780 piglets (154, 209, 189 and 228 from herds 1, 2, 3 and 4, respectively) were evaluated in the study on ADG.

Apart from diarrhoea, only a few disease problems were recognised during the daily examinations of the herds. However, arthritis was rather prevalent in herd 4 (n = 19). Skin abrasions were the only extra-intestinal clinical symptoms sufficiently prevalent and evenly distributed between herds to be included in the statistical evaluations.

On the day of birth, approximately 20% of the piglets did not have any faeces in rectum at the time of examination. On the other days of the study absence of faeces was observed in 10% of the piglets. In both the diarrhoeic and the non-diarrhoeic piglets the colour of faeces was most often (in 70-80% of piglets) yellow. Neither debris nor blood was evident.

Table 1 presents the daily prevalence of diarrhoea within each herd. The prevalence of diarrhoea in herd 1 was high (around 30%) on each day of the study. In the other herds, the prevalence was high on the first day of life, but otherwise markedly lower than in herd 1. A total of 229 piglets (26%) were diarrhoeic on the day of birth. Within this group, 50% were diarrhoeic on the day of birth exclusively (data not shown).

**Table 1:** The prevalence of diarrhoea on each day of the study within and across herds

**Herd**	**Day 1**		**Day 2**		**Day 3**		**Day 4**		**Day 5**	
	**n**^**1**^	**D**^**2**^**(%)**	**n**	**D (%)**	**n**	**D (%)**	**n**	**D (%)**	**n**	**D (%)**
1	207	26	202	34	201	24	197	39	194	40
2	228	41	226	23	226	13	224	11	222	15
3	202	18	200	9	198	11	195	16	193	16
4	237	19	236	9	235	8	235	6	234	7
Across herds	874	26	864	18	860	14	851	18	843	19

^1^Refers to the number of piglets alive at the day in question. ^2^Diarrhoeic piglets (piglets having watery or liquid consistency of faeces).

Table 2 presents a descriptive summary of the diarrhoea seen in the four herds with respect to prevalence, ADG, and mortality. In herd 1, nearly half the piglets were diarrhoeic for more than one day, and the mortality among piglets being diarrhoeic (irrespective of diarrhoeal level) was approximately 25% vs. 6% among the non-diarrhoeic piglets. In the other herds, similar mortalities were seen in non-diarrhoeic piglets and piglets being diarrhoeic for one day. However, additional days of diarrhoea seemed to be associated with a higher mortality in herd 2 and herd 4. The duration of diarrhoea differed according to the parity of the sow. One third of the piglets born by first parity sows were diarrhoeic for >1 day, whereas this was observed for one fifth of the piglets born by mature sows only (data not shown).

**Table 2:** Descriptive data on the diarrhoea in the four herds

		**Days with diarrhoea**											
		**None**			**1 day**						**>1 day**		
					**During day 1**			**During day 2-5**					
**Herd**	**n**^**1**^	**n**	**ADG (mean, sd)**	**Dead (%)**	**n**	**ADG (mean, sd)**	**Dead (%)**	**n**	**ADG (mean, sd)**	**Dead (%)**	**n**	**ADG (mean, sd)**	**Dead (%)**
**1**	207	49	186 (83)	6	15	221 (90)	27	49	158 (80)	22	94	115 (74)	27
**2**	228	88	179 (65)	6	48	194 (61)	2	33	165 (60)	6	59	169 (61)	12
**3**	202	106	185 (60)	8	22	193 (74)	5	43	167 (57)	5	31	156 (72)	6
**4**	237	150	190 (62)	2	30	190 (65)	3	32	147 (61)	6	25	158 (52)	12
**In total**	874	393	186 (65)	5	115	196 (67)	6	157	160 (65)	11	209	145 (71)	18

^1^This number refers to the number of piglets at day 1 of the study. ADG has only been calculated for the ones surviving until day 10 of life (780 in total).

On the fifth day of life, a total of 842 piglets were alive and examined for clinical signs of failure to thrive. Results are presented in Table 3. Signs of dehydration were only found in diarrhoeic piglets. Hollow flanks, protruding ribs, dull hair coat were more common in diarrhoeic piglets, than in pigs without diarrhoea. All clinical signs were more prevalent in pigs experiencing diarrhoea for more than one day. The general clinical picture of piglets suffering from diarrhoea was that of failure to thrive and wasting.

**Table 3:** Clinical signs of failure to thrive in 842 piglets on day five of life

**Days with diarrhoea**				
**Clinical finding**^**1**^	**None**	**1 day**		**>1 day**
		**During day 1**	**During day 2-5**	
Hollow flanks	18%	10%	23%	38%
Protruding ribs	3%	2%	9%	22%
Dull hair coat	38%	27%	41%	50%
Dehydration	0%	0%	3%	15%

^1^Definitions used: Hollow flanks: The area behind the ribs deviated inwards; Protruding ribs: Ribs and spine were visible; Dull hair coat: Hair coat appeared dull and longer than normal; Dehydration: sunken eye-balls and lack of skin-elasticity.

**Figure 1:**
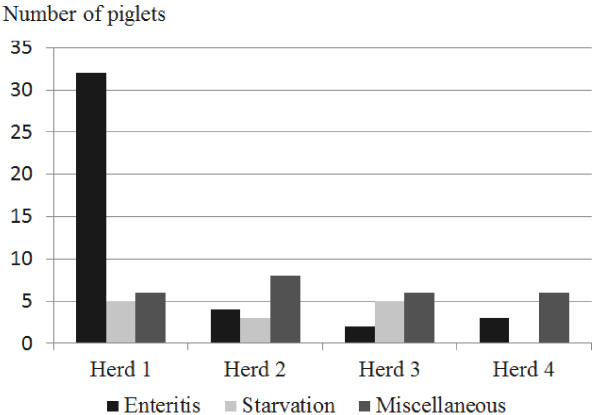
**Primary diagnoses at necropsy in 43, 15, 13 and 9 piglets from herd 1, 2, 3 and 4, respectively.** The following criteria were used to assign piglets to the respective diagnoses: Enteritis: Intestinal hyperaemia, flaccidity or mucosal lesions and liquid contents in colon. Starvation: Piglets were emaciated and stomachs and intestines were empty. Miscellaneous: All other diagnoses and cases without obvious findings.

In total, 80 piglets (9%) died within the first ten days of life. Mortality within herds 1, 2, 3 and 4 reached 43 (21%), 15 (7%), 13 (6%) and 9 (4%), respectively. The majority of deaths in herds 2–4 occurred during the first five days of life. In herd 1, deaths were evenly distributed throughout the first ten days of life. Figure 1 shows the primary diagnoses assigned at necropsy. Within herds 1 and 4, a few cases of enteritis (9 of 32 and 3 of 3 cases, respectively) exhibited fibrino-necrotic gross lesions. The remaining cases exhibited less pronounced lesions. In addition to the diagnoses of starvation shown in Figure 1, starvation was recorded as a secondary diagnosis in a total of five piglets.

### Weight gain

Results of the mixed linear model are presented in Table 4. As shown, both diarrhoea-associated variables (diarrhoea and Diarrhoeal Status of Litter (DSL)) had significant (P = 0.01 and P < 0.001) effects on ADG. Being part of a severely affected litter had a markedly larger effect (-38 g per day) than the individual status of the piglet (-9 - -14 g per day, depending on the duration of diarrhoea). Piglets that were only diarrhoeic on the day of birth were not negatively affected on ADG compared to the non-diarrhoeic ones. No confounding effects were recognised. The ICC estimated for the litter random effect was 42%, indicating a very large influence on weight gain by litter of origin. In pairwise comparisons, ADG in non-diarrhoeic piglets was significantly different from ADG in piglets having diarrhoea for >1 day. Also, ADG in piglets only being diarrhoeic at the day of birth was significantly different from ADG in piglets having diarrhoea for >1 day. Data were insufficient to show other statistically significant differences.

**Table 4:** Final linear mixed model on ADG in 780 piglets from the four herds

**Risk factor**	**Estimate (g/day)**	**SE (g/day)**	**P-value**
Intercept (1000 g birth weight)	151	7	
Parity			0.7^1^
Mature	0		
Young	5	11	
Birth weight (per 100 g increase)	9	0.6	< 0.001
Diarrhoea^2^			0.01
None	0^a^		
1 day (during day 1)	4^a^	6	
1 day (during day 2–5)	-9^ab^	5	
>1 day	-14^b^	5	
Diarrhoeal status of litter			< 0.001
Mildly affected	0		
Severely affected	-38	11	
Random effect litter^3^	42%		

^1^Parity was kept in the model due to its biological importance.

^2^Levels of diarrhoea with different letters as superscript are significantly different (α < 0.05) when compared pairwise.

^3^The random effect of litter is displayed as the Intraclass Correlation Coefficient (ICC).

### Mortality

Results of the generalized linear mixed model are presented in Table 5. None of the diarrhoea-associated variables (diarrhoea or DSL) were kept in the final model, indicating that diarrhoea was not a significant risk factor with respect to mortality, when other variables and random litter effects were taken into account. Herd of origin was the most important risk factor in the model. In herd 1, odds for dying were significantly higher than in the other herds and almost twelve times as high as in herd 4, which had the lowest odds for dying. No confounding effects were recognised. In pairwise comparisons, herd 1 was the only herd significantly different from the others.

**Table 5:** Results of the final generalised mixed model on mortality during the first 10 days of life in 874 piglets from the four herds

**Risk factor**	**Coefficient**	**SE**	**OR**^**1**^	**P-value**
Intercept (1000 g birth weight)	-3.9			
Herd^2^				< 0.001
Herd 4	0^a^			
Herd 3	1.0^a^	0.7	2.3	
Herd 2	0.5^a^	0.7	1.3	
Herd 1	2.7^b^	0.6	11.8	
Parity				0.06^3^
2nd-7th	0			
1st	0.8	0.4	1.8	
Birth weight (per 100 g increase)	-0.3	0.06	0.6	<0.001
Gender				0.001
Female	0			
Male	0.9	0.3	2.1	
Random effect of litter^4^	29%			

^1^The OR presented is the population average OR, and relates to an average piglet in any litter of the study.

^2^Herds with different letters as superscript are significantly different (α < 0.05) when compared pairwise.

^3^Parity was kept in the model due to its biological significance.

^4^The random effect of litter is displayed as the Intraclass Correlation Coefficient (ICC).

## Discussion

The study monitored the daily faecal consistency in individual neonatal piglets, and therefore offered an opportunity to thoroughly evaluate prevalence, timing and duration of NNPDS, and its effect on piglets. The observational character of the study was, however, affected by the removal of 110 piglets for diagnostic purposes. This interference, which potentially increased ADG and decreased mortality in litters supplying piglets for diagnostic purposes, was addressed by inserting a random effect of litter in the statistical models. Altogether, we considered that taking out piglets for laboratory examination during the course of the study was the best way to obtain a microbiological diagnosis of the existing clinical problems.

The procedure of adjusting litters to only 11 or 12 piglets and excluding the smallest piglets aimed at avoiding insufficiency of colostrum and milk. Milk-filled stomachs at necropsy generally confirmed, that diarrhoea due to starvation was rare during the period of investigation. Under normal field conditions, starvation is likely to be more prevalent and to interfere with the clinical picture of the syndrome to a higher extent.

Antibiotic treatment was allowed due to ethical concerns but potentially influenced the results of the study. However, since the syndrome seems to be characterised by non-responsiveness to antibiotics (personal communication S.E. Jorsal, National Veterinary Institute, Technical University of Denmark) the beneficial effects of antibiotics on ADG and mortality were probably relatively small.

A relatively large group of piglets (10-20%) did not have faeces in rectum at the daily examinations and consequently were recorded as non-diarrhoeic. Therefore the daily prevalence of diarrhoea (ranging from 6% to 40%) might have been underestimated. For the analysis, this was not considered a major problem since diarrhoeal status of each piglet was evaluated for the total study period.

Clinical signs of failure to thrive were overt in piglets being diarrhoeic for >1 day, and in some cases even 1 day of diarrhoea resulted in hollow flanks, protruding ribs and signs of dehydration. These findings match experiences from swine practitioners, that NNPDs is a debilitating syndrome, significantly affecting the well-being of piglets (personal communication, S.E. Jorsal).

The negative effects of diarrhoea estimated in this study were comparable to the -8 g per day estimated in a previous study involving the whole suckling period [8]. In the current study, even piglets only being diarrhoeic for a single day (if this was not the day of birth) had a reduced ADG of 9 g compared to non-diarrhoeic piglets. The measurable (though not statistically significant) effect of a single day with diarrhoea underlines the severity of the syndrome.

Interestingly, many piglets (26%) were diarrhoeic on the day of birth and those (50%) that were only diarrhoeic on the day of birth were not negatively affected on ADG. Apparently, many cases of diarrhoea on the day of birth were unrelated to disease.

The large effect of DSL pointed out that the diarrhoea seen on the level of litters was the most important kind of diarrhoea in terms of disease. ADG was not affected by herd of origin, but was heavily influenced by litter of origin (ICC = 42%). A large litter effect was not surprising since it comprised both issues related to the study design (as discussed earlier) and all factors related to the performance of the individual sows. Lack of herd effect on ADG was in accordance with the study by Johansen et al. [8].

Compared to the study by Svensmark et al. [7], which estimated the effect of diarrhoea at the litter level to be -14 g per day, the litter-related estimate of -38 g in this study was more pronounced. Possibly, NNPDS affected the piglets more violently than the diarrhoea seen in the previous study. However, the estimates from the two studies were not directly comparable, since the former included the whole suckling period and relied on farmers registrations of diarrhoea.

Regarding mortality, herd was the most important risk factor, with odds for dying in herd 1 being 5–12 times higher than in the other herds. Descriptive data indicated that the excess mortality seen in this herd was caused by diarrhoea, since the mortality among diarrhoeic piglets, irrespective of the duration of diarrhoea, was four times higher than the mortality among non-diarrhoeic ones. In the other herds, mortalities were lower at all levels of diarrhoea, and any association between diarrhoea and mortality was less obvious.

Suckling piglet diarrhoea has often been associated with mortality. In a previous study involving 3600 piglets from a single herd, neonatal diarrhoea was estimated to increase odds of dying by 2.7 [9]. Another study involving piglets from 70 herds showed that diarrhoeic litters had increased losses during the suckling period of 0.8 piglets [10]. Furthermore, different studies have diagnosed enteritis as the primary cause of death in 4-14% cases of suckling pig mortality [11-13].

In the current study, we did not find an effect of NNPDS on mortality when herd of origin and other risk factors were taken into account. The underlying aetiology of diarrhoea in the study by Gardner et al. [9] was not investigated, and discrepancies could perhaps be explained by differences in aetiology. Herd effects were not considered in the results presented by Lingaas et al. [10], however, may have explained some of the losses attributed to diarrhoea. Overall, it is important to notice that the generally low mortality in the current study was probably partly due to study design issues. Despite these limitations, the study indicated that NNPDS was generally not associated with high mortality. This finding matched herd experiences as reported by swine practitioners [14].

## Conclusions

The diarrhoeas observed within herds suffering from NNPDS were yellow with no evidence of blood or debris. Three of the four herds had similar courses of NNPDS. In these herds the daily prevalence of diarrhoea was approximately 15%, deaths occurred within the first five days of life, and the mortality was generally low. The most prevalent diagnoses assigned at necropsy were miscellaneous and starvation. In the last herd (herd 1), diarrhoea affected more piglets, and many piglets were affected for a longer period of time. In this herd, a high mortality was seen during the whole study period, and enteritis was the most prevalent diagnosis at necropsy.

Overall, the study showed that NNPDS severely affected the well-being in piglets and reduced the ADG. Data were not sufficient to estimate a significant effect of diarrhoea for a single day, however, a tendency was clear, and this finding underlines the severity of the condition. Diarrhoea restricted to the day of birth was common and did not affect ADG. Thus, the study suggested this phenomenon to be unrelated to the syndrome.

Since the diarrhoeal status of the litter had important effects on ADG, future research on NNPDS should probably focus on piglets in severely affected litters.

Overall, the study demonstrated that NNPDS was not associated with mortality. In the herd associated with the highest odds for mortality, however, the increased mortality appeared to be due to diarrhoea.

## Methods

### Study design and inclusion of herds

The study was an observational cross-sectional study with follow-up, carried out in four Danish sow herds. Herds were included based on five criteria; problems with diarrhoea during the first week of life with a poor response to antibiotics (≥30% diarrhoeic litters for a period of ≥ 6 months), routine vaccination of sows against *E. coli* and *C. perfringens* type C, failure of preventive management interventions as verified by the local veterinarian, a PRRS negative farrowing unit as demonstrated in blood samples and negative results of routine diagnostic examinations for ETEC, *C. perfringens* type C and rotavirus A in five diarrhoeic piglets aged one to four days. During the study period, approximately 30 piglets per herd were taken out for microbiological examination. The examinations carried out indicated that the herds did not suffer from known infectious causes of diarrhoea and therefore supported a diagnosis of NNPDS [6].

### Practical setup in the herds

Litters (approximately 20 litters from one farrowing batch per herd) were standardized to 11 or 12 piglets by simple random sampling among littermates with a minimum birth weight of 800 grams. No cross-fostering was allowed.

As a general rule no antibiotics were given on the first two days of life. In herd 4, however, eight pigs were treated on the second day of life due to arthritis. From the third day of life and onwards, diseases were treated with antibiotics according to individual herd routines. As a result of this, most of the diarrhoeic piglets in the study were medicated. Non-antibiotic oral supplements were allowed to be used according to individual herd routines. In herd 2 and 4 a milk formula was given to approximately 10 piglets per herd for one or two days. In herd 1 and 3, no oral supplements were used.

### Examination of piglets

Piglets were weighed at birth and at ten days of life. In each piglet rectal swabs were evaluated on a daily basis from the day of birth and five days forwards. On each evaluation, consistency of faeces was categorized into one of six categories; No faeces present (the rectal swab was dry and clean), watery, liquid, creamy, firm or solid (solid bulbs on the rectal swab). For the analyses, a piglet was defined as diarrhoeic on a particular day if having watery or liquid consistency of faeces. Microbiological testing was not performed on piglets in the study.

During the first five days of life, registrations on arthritis, respiratory disease, CNS-related disease and skin abrasions on head and fore-knees were carried out. Piglets were registered with the respective diagnoses if registered on any day. The presence of hollow flanks, protruding ribs, dull hair coats and dehydration (sunken eye-balls and loss of skin-elasticity) was registered on the fifth day of life as clinical signs of failure to thrive due to diarrhoea.

During the 10 day study period, all deaths were individually recorded and necropsies were performed on all piglets dying. At necropsy, piglets were assigned to one of three primary diagnoses; enteritis, starvation or miscellaneous. A diagnosis of enteritis was assigned if hyperaemia, flaccidity or mucosal lesions were seen in the intestines and contents of colon were liquid. A diagnosis of starvation was assigned if the piglet was emaciated, stomach and intestines were empty and no other findings were obvious. Cases of crushing, constipation, castration injuries as well as cases with no obvious lesions were assigned to the miscellaneous category. In cases when empty stomachs were seen in association with other lesions, starvation was assigned as a secondary diagnosis.

All procedures in the herds were carried out by the corresponding author.

### Description of variables

#### Outcome variables

Weight gain (measured in g per day) and death (yes/no) during the period from birth until ten days of age, were the dependent variables in the two models of the study.

#### Explanatory variables

The explanatory variable of primary interest was diarrhoea which was initially individually recorded on a daily basis. Subsequently, based on the duration and timing, four levels of diarrhoea were defined; “None”, “1 day (during day 1)”, “1 day (during day 2–5) and “>1 day” (see Table 6). These levels were introduced in order to be able to distinguish between diarrhoea at the day of birth and diarrhoea of different duration. On the litter-level, the variable “Diarrhoeal Status of Litter (DSL)” was introduced, in order to dichotomise litters into mildly affected litters (<50% diarrhoeic piglets) vs. severely affected litters (≥50% diarrhoeic piglets). Table 6 presents all explanatory variables addressed in the statistical models of the study.

**Table 6:** Explanatory variables addressed in the models on ADG and mortality

**Explanatory variable**	**Level**	**Interpretation of levels**
**Primary**		
Diarrhoea	None	The piglet was not diarrhoeic at any day
	1 day (during day 1)	The piglet was diarrhoeic for one day - at the day of birth
	1 day (during day 2–5)	The piglet was diarrhoeic for one day during the second to fifth day of life
	> 1 day	The piglet was diarrhoeic for more than one day during the five day study-period
**Secondary**		
**Litter level**		
Parity	Young	1st parity
	Mature	2nd – 7th parity
Diarrhoeal status of litter (DSL)	Severely affected	50% or more of the piglets in the litter were diarrhoeic for one or more days (the day of birth did not count)
	Mildly affected	Less than 50% of the piglets of the litter were diarrhoeic for one or more days (the day of birth did not count)
**Piglet level**		
Gender	Male	
	Female	
Birth weight	Continuous scale	
Skin abrasions	Yes	Skin abrasions on head or fore-knees at any point during the study period
	No	No skin abrasions on head or fore-knees
**Herd effect**	1	Effect of being born in herd 1
	2	Effect of being born in herd 2
	3	Effect of being born in herd 3
	4	Effect of being born in herd 4

#### Statistical analyses

For the study on weight gain, a linear mixed-effect model was used, whereas a generalised linear mixed-effect model was used in the study on mortality. Both models were fit in R [15] using the lme4 package [16]. The assumption of linearity in the linear mixed model was verified by visual inspection of the xy-plot of ADG against birth weight. The linearity of birth weight at the log odds scale for mortality in the generalised linear mixed model was assessed by transforming birth weight into a categorical variable based on quartiles and then verify a decreasing trend of the estimates for the levels of the categorical variable.

In the models, herd of origin was included as a fixed effect, since we were interested in the specific effect of each herd. Litter of origin was included as a random effect to correct for clustering within litters. All secondary risk factors and all possible two-way interaction terms with diarrhoea were included in the initial models. Model reduction was carried out using stepwise backwards elimination, removing variables with p > 0.05. Since parity was considered of overall biological importance, this variable was forced into the models. Confounding was assessed by re-entering variables into the final models and checking if estimates for diarrhoea changed. Pairwise post-hoc comparisons within significant variables with more than two levels were carried out using the lsmeans package in R [17].

### Ethical approval

The study was conducted in accordance with the guidelines of the Danish Ministry of Justice with respect to animal experimentation and care of animals under study. Clinical examinations and weighing were carried out with consideration to the welfare of the pigs by a skilled person (HK).

## Competing interests

The authors declare that they have no competing interests.

## Authors’ contributions

All authors contributed to the design of the study. Inclusion of herds, clinical examination in the herds and statistical analyses were performed by HK. All authors participated in drafting the manuscript and proofreading of the final manuscript. All authors read and approved the final manuscript.
